# Editorial: Chemical-physical interactions in bitumen: towards environmentally sustainable road materials

**DOI:** 10.3389/fchem.2026.1933157

**Published:** 2026-07-28

**Authors:** Valeria Loise, Pietro Calandra, Valeria Butera, Paolino Caputo, Bagdat Teltayev

**Affiliations:** 1 Department of Chemistry and Chemical Technologies, University of Calabria, Rende, Italy; 2 CNR-ISMN, National Council of Research, Institute for the Study of Nanostructured Materials, Roma, Italy; 3 Department of Science and Biological Chemical and Pharmaceutical Technologies, University of Palermo, Palermo, Italy; 4 U. Joldasbekov Institute of Mechanics and Engineering, Almaty, Kazakhstan

**Keywords:** asphalt, bitumen, chemistry - properties relationship, interactions, sustainability

Road infrastructure forms the backbone of modern society, supporting economic development, mobility, and regional connectivity. At the heart of this infrastructure lies bitumen, a complex hydrocarbon-based viscoelastic fluid that has served for decades as the principal binder of crushed stones in asphalt pavements. Despite its widespread use and proven performance, the demand for longer-lasting pavements as well as the need for limiting the use of petroleum-based materials, or even their replacement with green alternatives, continue to challenge conventional bituminous materials. Consequently, improving the sustainability of asphalt pavements has become one of the foremost priorities in contemporary pavement engineering.

Traditionally, the enhancement of asphalt performance has relied on the modification of neat bitumen through the incorporation of polymers, fibers, nanoparticles, bio-based materials, and various chemical additives. These modifiers have demonstrated significant potential in improving rutting resistance, fatigue performance, low-temperature cracking resistance, moisture susceptibility, and ageing characteristics. However, the effectiveness of these modifications is fundamentally governed by the physicochemical interactions occurring at the molecular and microstructural levels. Understanding these interactions is therefore essential for the rational design of next-generation bituminous materials.

To address these challenges, an interdisciplinary perspective extending beyond the traditional boundaries of pavement engineering is needed. In this regard, while bitumen has historically been studied primarily from an engineering standpoint, advances in chemistry, physics, materials science, computational modeling, and nanotechnology have revealed the complexity of bituminous systems. The colloidal nature of bitumen, its diverse chemical constituents, and its interactions with modifiers require analytical approaches capable of linking molecular phenomena with macroscopic engineering performance. Ultimately, these cross-disciplinary insights pave the way for designing environmentally sustainable road materials.

The present Research Topic of papers is a small selection from this continuously expanding and developing subject.

Yu et al. addressed the problem of poor adhesion between the inorganic binder and the RAP (Reclaimed Asphalt Pavement) aggregate caused by the asphalt film on the surface of the RAP itself. The authors therefore propose modifying the RAP interface by applying a mineral admixture paste coating. This approach improves the mechanical properties and environmental impact of recycled mixtures. The authors highlighted that modifying the interface leads to significant improvements in compaction, indirect tensile strength, drying shrinkage, and even carbon emissions. In the latter case, with 28% modified RAP, carbon emissions per unit volume are reduced by as much as 54.3% compared to cement-stabilized crushed stone.

In a parallel effort, Chen et al. focused on the morphological variability of natural aggregates, which compromises test reproducibility. To address this, they developed a protocol to produce high-performance cementitious artificial aggregates (ACA) using 3D printing and mortar molds. The authors analyzed their research with great intellectual integrity, noting that the morphological uniformity of the ACAs successfully reduced the coefficient of variation in fatigue tests and maintained Z-scores within 2 in a 7-laboratory interlaboratory study. However, they identified three key weaknesses: the study relied on single-factor experiments, lacked long-term durability data, and omitted a comprehensive assessment of inter-operator variability. Consequently, the authors suggest that further studies are needed to provide a more complete overview.

Ashimova et al. aimed to improve asphalt’s mechanical properties—including compressive strength, rutting, cracking, and thermal resistance—using EVA granules and recycled crumb rubber. Using FTIR, they evaluated the interaction mechanisms between the bitumen and modifiers, observing enhanced thermal stability and deformation resistance. The authors identified distinct mechanisms of action: EVA structurally changes the binder by introducing polar functional groups into the matrix, whereas crumb rubber interacts purely physically. Ultimately, they highlight EVA granules as an effective, sustainable approach to boosting asphalt reliability and longevity.

Mendoza et al. investigated modifications involving graphene oxide (GO) under tropical service conditions. The authors begin by synthesizing GO using their own eco-friendly, aqueous method that avoids the strong chemical oxidation typical of Hummers-type procedures. They also found that GO leads to improved resistance to rutting and, above all, enhances the thermal stability of asphalt in tropical climates. However, since their study also focused on short-term ageing, the investigations showed that GO does not reduce ageing-induced stiffening. Consequently, the authors suggest that further investigations into the kinetics of ageing using PAV are necessary. It should be noted, however, that the operating range identified by the authors is 0.2%–0.4% of GO.

Like graphene, other nanomaterials also improve the high-temperature properties of bitumen. Fan et al. investigated how bitumen modified with SBE and nano-TiO_2_ react to two repeated ageing/rejuvenator cycles. In general, modified asphalt and asphalt that has undergone a single regeneration are suitable for conventional pavements or those subject to medium traffic loads, which require balanced performance at both high and low temperatures and fatigue resistance. By contrast, modified asphalt that has been aged (either once or twice) and regenerated for a second time is more suitable for pavements subject to heavy loads and high temperatures, or for special sections with less stringent low-temperature performance requirements.

The study conducted by Alfé et al. explores the use of pyrolysis oil derived from recycled tyres to regenerate asphalt with a view to the circular economy and sustainability. The oil used is a mixture of aromatic compounds, of which limonene is the most abundant, followed by certain alkylbenzene derivatives. The aromatic composition of the oil is crucial to its regenerative effect, as it promotes interaction with asphaltene structures. Regeneration occurs through the breakdown of asphaltene aggregates and the restoration of the self-assembling structure. The research has therefore shown that pyrolysis oil from recycled tyres is an effective regenerant for aged asphalt.

Hu et al. have proposed a study aimed at both mitigating the drawbacks of using natural diatomite and improving its efficiency, whilst providing practical guidance on regulating its content during production. To this end, they investigated the effect of its chemical composition on the asphalt mixture water stability. Using molecular dynamics, the authors simulated the diatomite-asphalt interface to study the mechanism governing water stability performance. The research showed that by adjusting the chemical composition and microstructure of the interface, it is possible to significantly improve the bituminous mixture’s resistance to water damage and its durability, thereby extending the service life of the road and reducing maintenance costs.

The editors hope that they will convey the excitement, interest, enthusiasm, and drive of the “bitumen” community that is constantly innovating and responding to new, often formidable, challenges.

